# A 3.8 Å resolution cryo-EM structure of a small protein bound to an imaging scaffold

**DOI:** 10.1038/s41467-019-09836-0

**Published:** 2019-04-23

**Authors:** Yuxi Liu, Duc T. Huynh, Todd O. Yeates

**Affiliations:** 1 UCLA Department of Chemistry and Biochemistry, Los Angeles, CA 90095 USA; 2 UCLA-DOE Institute for Genomics and Proteomics, Los Angeles, CA 90095 USA; 3 UCLA Molecular Biology Institute, Los Angeles, CA 90095 USA; 4 California NanoSystems Institute, Los Angeles, CA 90095 USA

**Keywords:** Protein design, Cryoelectron microscopy

## Abstract

Proteins smaller than about 50 kDa are currently too small to be imaged at high resolution by cryo-electron microscopy (cryo-EM), leaving most protein molecules in the cell beyond the reach of this powerful structural technique. Here we use a designed protein scaffold to bind and symmetrically display 12 copies of a small 26 kDa protein, green fluorescent protein (GFP). We show that the bound cargo protein is held rigidly enough to visualize it at a resolution of 3.8 Å by cryo-EM, where specific structural features of the protein are visible. The designed scaffold is modular and can be modified through modest changes in its amino acid sequence to bind and display diverse proteins for imaging, thus providing a general method to break through the lower size limitation in cryo-EM.

## Abstract (web summary)

Proteins smaller than about 50 kDa are currently too small to be imaged at high resolution by cryo‐electron microscopy (cryo‐EM). Here authors design a protein scaffold that binds 12 copies of a small 26 kDa protein (GFP), which allowed visualizing GFP at a resolution of 3.8Å by cryo‐EM.

## Introduction

Methods for visualizing macromolecules in atomic detail have transformed our understanding of molecular biology. Yet the leading techniques—X-ray crystallography, NMR, and electron microscopy—all face obstacles that limit their universal application. X-ray crystallography presents difficulties in crystallization, while NMR methods become challenging for very large macromolecules. For cryo-EM, despite recent technological advances that have revolutionized the field (reviewed in ref. ^1,2^), a lower size limitation has prevented application of this powerful method to proteins smaller than about 50 kDa^3^, which is larger than the average cellular protein. Overcoming this lower size barrier could bring electron microscopy close to the ultimate goal of a universally applicable method for structural biology.

The goal of visualizing small proteins by cryo-EM has motivated research efforts along multiple directions. With optimal sample preparations it has been possible in a recent study to reach high resolution (better than 3 Å) for structures as small as 64 kDa^4^. In other recent studies, the use of phase plates^5^ have been important in enhancing image contrast. There, a resolution of 3.2 Å was achieved for human hemoglobin (64 kDa) and streptavidin (52 kDa)^6,7^. These studies on small proteins have begun to approach the 38 kDa theoretical lower limit proposed in 1995^3^. An alternative approach is to design molecular scaffolding systems—i.e. molecules of known structure that are large enough to visualize in atomic detail by cryo-EM, and simultaneously able to bind and display a smaller protein molecule of interest, effectively making the smaller ‘cargo’ protein part of a larger assembly that can be structurally elucidated as a whole^8–12^. Two key challenges have hindered the development of useful cryo-EM scaffolds: rigidity and modularity. The cargo protein must be bound and displayed rigidly so that it does not become smeared out during reconstruction of the full structure. And to be practical the scaffold must be able to bind diverse cargo proteins with minimal re-engineering efforts.

**Fig. 1:**
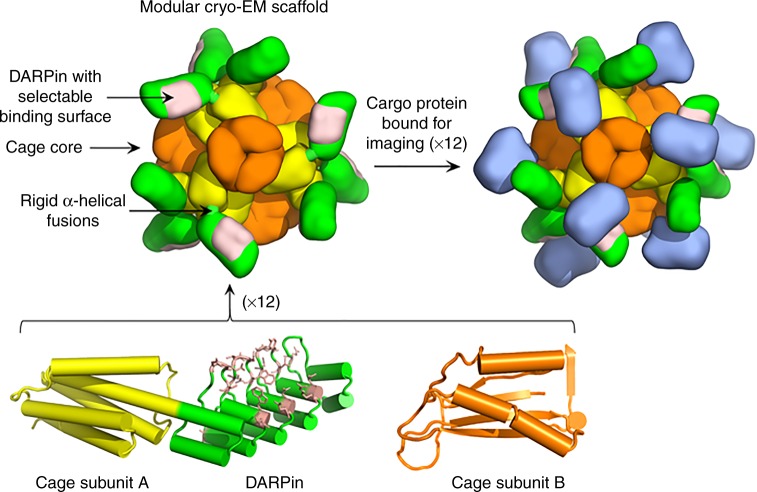
The design of a modular protein scaffold for cryo-EM imaging. The designed core assembly is composed of 12 copies of two protein subunits, A (yellow) and B (orange), in a tetrahedrally symmetric arrangement. Subunit A is genetically fused by a continuous alpha helical linker to a DARPin (green). Amino acid mutations (based on random library selection experiments) are inserted into the DARPin binding surface (pink) to confer tight binding of a cargo protein (blue) for cryo-EM imaging

In a recent work, we developed a scaffold that addresses the requirements for rigid display and modularity, while also exploiting the advantage of high symmetry to mitigate the common problem of preferred particle orientation in cryo-EM^11^. Our scaffolding system uses a DARPin (Designed Ankyrin Repeat Protein) as an adaptor component that is genetically fused by a continuous alpha helical connection to a central core comprised of a designed symmetric protein cage with cubic symmetry (12 orientations in symmetry T, Fig. 1)^13^. In prior work, DARPins have been developed as a facile framework for sequence diversification and selection, by phage display or other laboratory evolution methods, for binding to a wide range of target proteins^14–21^. With these ideas put together, our scaffold presents multiple symmetrically disposed copies of an adaptor protein whose sequence can be mutated to bind diverse cargo proteins for imaging. In our earlier structural study on this scaffolding system, referred to as DARP14, we analyzed the scaffold by itself without cargo bound and demonstrated that the DARPin adaptor component could be visualized by cryo-EM at a resolution ranging from 3.5 to 5.5 Å^11^.

Here we use our designed scaffold to bind a small cargo protein for imaging. After mutating the modular scaffold in order to bind green fluorescent protein (GFP), cryo-EM analysis is able to reveal structural details of the cargo protein. Remaining flexibility challenges and prospects for reaching even higher resolution are discussed.

## Results

### Modular cargo binding

As a first test case for our scaffolding system, we chose the 26 kDa green fluorescent protein (GFP) as a cargo molecule. GFP has been well-studied and DARPin sequences that bind various forms of GFP have been published^19,20^. Our current study employed the superfolder variant (sfGFP)^22^ with a V206A mutation. Compared to the prior study, we modified the DARP14 scaffold as follows. Motivated by the finding that the terminal repeats of the DARPin were more flexible and showed a compromised resolution, we adopted a set of mutations previously characterized to stabilize this region of the DARPin^23^. Finally, to convert the scaffold to a form that would bind GFP—and thus illustrating the modularity of the system—we exchanged the amino acid sequences in the binding loops on the original DARP14 scaffold with a sequence known as 3G124^19,20^. Taken together, these modifications make a symmetric scaffold that is specific to GFP. After binding GFP to the scaffold, the complex was purified and found by quantitative amino acid analysis to have nearly complete saturation of the binding sites on the scaffold; i.e. nearly 12 copies of GFP on each cubically symmetric scaffold. Negative stain EM showed assembled particles of the expected size and symmetry (Supplementary Figure 1).

### Single-particle cryo-EM analysis

**Fig. 2:**
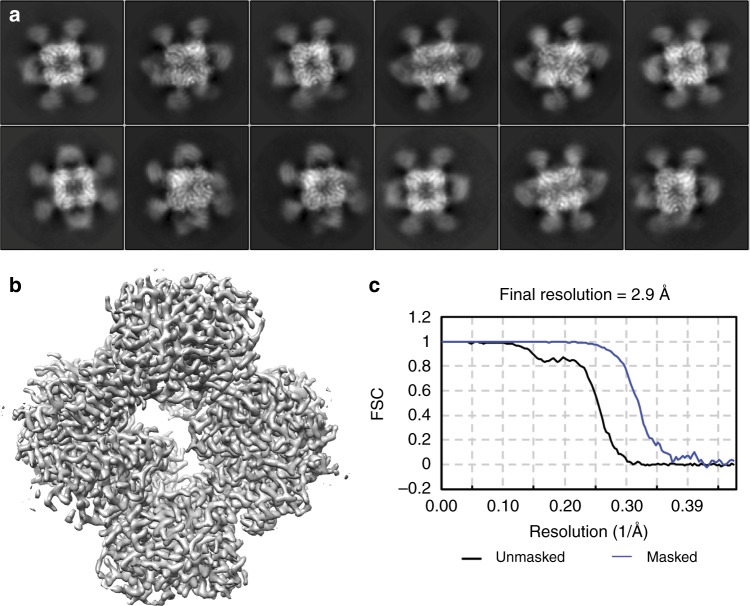
Cryo-EM data on GFP bound scaffolds. **a** 2D class images of the particles show strong features for the tetrahedral core along with clear but more diffuse density for the DARPin and the bound GFP cargo. The image box size in **a** corresponds to 271 Å. The 3-D density reconstructed only around the core structure (**b**) and the corresponding gold-standard Fourier shell correlation (FSC) curves (**c**) are shown for masked (blue) and unmasked (black) maps

To analyze the three-dimensional structure, we collected 1929 movie images of this complex under cryogenic conditions using a Titan Krios (Supplementary Figure 1). After initial data processing, 81,319 particles were selected for 3D analysis based on their 2D class averages (Supplementary Figure 2). The 2D class averages showed strong density for the symmetric core of the scaffold. Moreover, there was clear additional density for the DARPin adaptor and the bound GFP (Fig. 2a, compare to Fig. 2b in ref. ^11^). Some flexibility was evident for the DARPin and the GFP cargo. The density in these regions was generally weaker and more diffuse, indicative of multiple conformations and calling for special attention in the later analysis. Structural analysis in three dimensions made it possible to assess further the degree of flexibility vs rigidity imposed on the cargo protein by the scaffold.

**Fig. 3:**
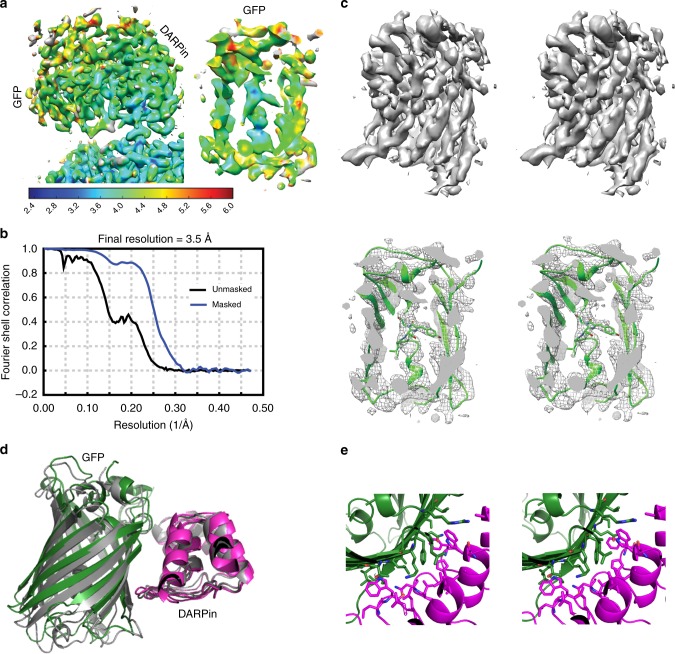
Near-atomic resolution map of the DARPin adaptor and its bound cargo protein GFP. **a** A density map obtained following multi-body refinement in RELION is shown colored by local resolution in a side view (left) and a sliced view (right), showing the central helix in the center of the GFP beta barrel. **b** Gold-standard FSC curve for the reconstruction. **c** Two wall-eyed stereo views of the sharpened GFP density map. The full GFP density is shown at the top; a sliced view with a fitted GFP atomic model is shown at the bottom. The fluorophore and Leu 64 (shown in sticks) in the central helical region have clear surrounding density. **d** Comparison between the DARPin plus GFP model in this study (GFP in green, DARPin in magenta) and the previously reported 3G124 eGFP crystal structure (grey, PDB 5MA8). The two structures are aligned on the DARPin portion. **e** Wall-eyed stereo views of the extensive interface between the DARPin (magenta) and GFP (green). The side chains of the bulky residues forming the interface are shown in sticks

An initial 3D image reconstruction was performed using the cryoSPARC program^24^, focusing on the symmetric core of the scaffold with enforced T symmetry (Fig. 2b, Supplementary Figure 2). This map exhibited an overall resolution of 2.9 Å with good amino acid side chain features all around (Fig. 2b, c). Refining the structure of the scaffold core against this map showed that the core structure is largely undisturbed relative to our previous cryo-EM model, with an overall RMSD of 0.90 Å between the two^11^. To reconstruct the attached DARPin and GFP components, the particles were then passed to the RELION program^25,26^ to perform signal subtraction and masked classification^27,28^. After expanding the particles by T symmetry and subtracting away the density corresponding to 11 of the 12 DARPin adaptors and their bound GFP molecules, the remaining density was classified again in 3D without alignment. Interestingly, about 13.3% of the particles have very weak densities for the adaptor and cargo, likely indicating that these instances suffered from very different (i.e. bent) orientations for the protruding components. Three classes had good density for the DARPin and the bound GFP components, with slight differences in orientation for the distinct classes. They showed density around the previously observed secondary contact point between the DARPin and the scaffold core (Gly 187 on DARPin and Gly 108-Thr 109 on subunit A of the scaffold core, Supplementary Figure 3). We performed two sequential masked refinements on particles from class 1, followed by a multi-body refinement. The masks used in the refinements increasingly focused on a single DARPin and its bound GFP (Supplementary Figure 2). The multibody refinement searched the DARPin and GFP orientation locally while being constrained by its position relative to the symmetric core. The final reconstruction reached 3.5 Å in overall resolution for the body containing the DARPin and GFP, with the scaffold core at a somewhat higher resolution than the DARPin or GFP (Fig. 3a–d).

### Cargo imaging and resolution

Our result showed that the flexibility present in the DARPin and bound cargo (GFP) can be mitigated with established data processing technique, as described in Methods. The resolution that was ultimately achievable for these components was remarkably good. The local resolution for the DARPin has a median value of 3.55 Å (with a 1st to 3rd quartile range of 3.3–4.05 Å)^29^. This represents a notable improvement over our initial report on the scaffold by itself, likely due to the stabilizing mutations introduced into the terminal repeat of the DARPin and the presumed stabilizing effects of binding to the cargo protein, GFP. The local resolution for the bound GFP has a median value of 3.8 Å (with a 1st to 3rd quartile range of 3.55–5.55 Å) (Fig. 3a). Key structural features of the protein are clear. The side of GFP contacting the DARPin shows a better resolution than the outward-facing side. In the regions of better resolution, the slanting beta strands in the GFP beta barrel are separate and clearly traceable, and side chain densities for some of the bulky residues are also visible. The alpha helical segment passing through the center of the β-barrel exhibits a local resolution of 3.5 Å, with side chain and fluorophore features clearly visible in the sharpened density map (Fig. 3c, Supplementary Movie 1). This level of detail represents a benchmark for imaging the structures of scaffolded small proteins by cryo-EM.

We undertook calculations to evaluate how much structural information could be extracted at the current resolution of 3.8 Å. It was possible to refine the DARPin with secondary structure restraints based on a reported unbound structure (PDB 6C9K) and to refine GFP as a rigid body based on a known crystal structure (PDB 4W6B). Refinement revealed that the individual ankyrin repeats of the DARPin adaptor expanded slightly further away from each other on the side distal from the bound GFP. In addition, our bound superfolder GFP is slightly rotated compared to the eGFP bound to a DARPin with the same loop sequence (PDB 5MA8, Fig. 3d). Overall, the final model shows a good fit to the map density, with clear chain tracing in much of the structure, and agreement with side chain density in the higher resolution regions (Fig. 3c). Density is evident for amino acid side chains at the GFP-DARPin interface. The extensive nature of those interactions, which are important for presenting the cargo protein in a well-defined orientation, are illustrated in Fig. 3e.

## Discussion

Our results illustrate a route for overcoming the critical lower protein size limit in cryo-EM. While the current resolution allows for visualization of useful structural features of a protein, it falls just short of providing the atomic detail required to elucidate the structures of novel proteins. That goal will likely be possible by improving the resolution by about 0.5 to 1 Å, to reach the 3 Å range. Our scaffolding system offers strategies for realizing such an incremental improvement. In particular, the high symmetry of the system admits additional design components for introducing stabilizing connections between symmetry-related pairs of the protruding DARPin components. Only modest advances will be needed in order to visualize atomic detail for small proteins. The value of the DARPin system is also demonstrated by another recent report of an engineered scaffolding system based on the D2 tetrameric aldolase utilizing a DARPin as the adaptor to image eGFP, albeit at a lower resolution (5–8 Å)^12^. Not only is GFP reconstructed to higher resolution in our study, but our designed scaffold provides higher symmetry (T), with concomitant advantages in mitigating the problem of preferred particle orientation.

Among the varied strategies under development for overcoming the lower size limit in cryo-EM, scaffolding approaches are likely to offer advantages beyond simply increasing the effective size of the imaging target. When a highly symmetric scaffold is employed, as it is here, the frequently-encountered problem of preferred particle orientation diminishes. Even if a symmetric particle exhibits an uneven orientation distribution, its symmetrically repeating components are effectively viewed from multiple directions simultaneously. The presence of multiple copies of a cargo protein on each particle also assures that some will be away from the air-water interface, where structural integrity is often compromised due to poorly understood surface chemistry and mechanical forces^30,31^. Scaffold approaches should also ultimately provide advantages in streamlined cryo-EM data processing, as a large symmetric core greatly simplifies the critical problem of establishing accurate particle orientations. Besides seeking improvements in resolution by improving scaffold rigidity, future experiments that include more diverse targets will be important in order to demonstrate the versatility and imaging advantages conferred by cryo-EM scaffolding systems of the type described here. Further optimizations of the system will be needed to ultimately achieve high throughput structural biology goals.

## Methods

### Cloning and protein expression

The sequence of the scaffold used in this paper, DARP14-3G124Mut5 (see Supplementary Information), was designed by replacing the original DARPin sequence^11^ with the anti-GFP DARPin sequence, referred to as 3G124^20^, while adding the Mut5 mutations into the C-terminal repeat^23^. The mutations are A299P, I302L, S303A, L311I, I314V, L218A, N319A (as numbered in the DARP14 subunit A sequence). DNA fragments carrying the DARP14-3G124Mut5 sequences were purchased (Integrated DNA Technologies) and cloned into pET-22b vectors using Gibson assembly^32^. Superfolder GFP V206A (sfGFP V206A) was cloned with quick-change PCR (see Supplementary Notes for primer sequences) to introduce the V206A mutation on a pET-22b vector carrying the superfolder GFP gene^22^. All DNA manipulations were performed in *E. coli* XL2 Blue cells (Agilent). DARP14-3G124Mut5 and sfGFP V206A were expressed separately in *E. coli* BL21 (DE3) cells (New England Biolabs) in Terrific Broth at 20 °C overnight upon 1 mM IPTG induction at O.D. 0.6.

Upon collection of the cells, DARP14-3G124Mut5 and sfGFP V206A pellets were mixed at a 3:1 mass ratio, resuspended in resuspension buffer (50 mM Tris, 250 mM NaCl, 5 mM imidazole, 5% (v/v) glycerol, pH 8.0) supplemented with DNAse, lysozyme, and protease inhibitor cocktail (Thermo Fisher Scientific) and lysed together by sonication. Cell lysate was first cleared by centrifugation at 20,000 x g for 20 min and loaded onto a HisTrap column (GE Healthcare) pre-equilibrated with the same resuspension buffer. DARP14-3G124Mut5 bound with sfGFP V206A was eluted with a linear gradient to 500 mM imidazole. Upon elution, 5 mM DTT was added immediately. The eluted protein was concentrated and further purified by passing through a Superose 6 Increase 10/300 GL column (GE Healthcare), eluted with 10 mM Tris pH 7.5, 500 mM NaCl, 1 mM DTT, 1% (v/v) glycerol. Fractions were assessed by SDS-PAGE and negative stain EM for the presence of complete DARP14-3G124Mut5 cages. Bound sfGFP V206A was evident by the green color and the occupancy was estimated by amino acid analysis performed at the UC Davis Molecular Structure Facility based on a least-squares fit to amino acid abundances.

### Negative stain EM

The concentration of a 5 μL sample of fresh Superose 6 Increase eluent was adjusted to ~50 μg/mL, applied to glow-discharged 300 mesh formvar-carbon copper grids (Electron Microscopy Sciences) for one minute and blotted away. After two washes with filtered water, the grid was stained with 2% uranyl acetate for 30 s. Images were taken on a Tecnai T12 or a TF20.

### Cryo-EM data collection

Fresh fractions from the Superose 6 Increase column containing DARP14-3G124Mut5 bound with sfGFP V206A were pooled and concentrated to 2 mg/mL. The sample was diluted to 1 mg/mL and final buffer composition of 10 mM Tris pH 7.5, 500 mM NaCl, 1 mM DTT, 0.5% glycerol immediately prior to freezing. Quantifoil 200 mesh 1.2/1.3 copper grids (Electron Microscopy Sciences) was treated with 0.1% poly-lysine (Sigma-Aldrich) for 4–6 h prior to freezing and cleaned of excess poly-lysine by washing with filtered water three times. 2.5 μL of sample was applied to the grids without glow discharging and frozen using a Vitrobot Mark IV (FEI). 1,929 movies were collected on a FEI titan Krios microscope (Thermo Fisher) with a Gatan K2 Summit direct electron detector in counting mode with image shift at a pixel size of 1.07 Å, and defocus values around −2.5 μm. Movies with 40 frames were collected over 8 s with ~7.00 e^−^* A^−2^* s^−1^ dose rate.

### Cryo-EM data processing and model building

Raw movies were corrected for beam-induced motion using MotionCor2^33^ and the CTF estimation was performed with CTFFIND4 on non-dose weighted micrographs^34^. 2D class averages of manually picked particles were used as templates in auto-picking in RELION 2.0^25^. Autopicking yielded 91,809 particles, which were extracted from motion corrected, non-dose weighted micrographs that included frames 3–20. A total of 10,490 particles were removed from two rounds of 2D classification, the remaining particles were passed into cryoSPARC^24^. Two initial T symmetry enforced models were calculated de novo using ab initio reconstruction. The good class from this reconstruction was selected and fed into a homogeneous refinement in cryoSPARC with an auto-tightening mask that produced the 2.9 Å map of the symmetric core. These particles went through another round of 3D refinement for CTF and beamtilt in RELION 3.0. Using Chimera^35^, densities corresponding to 11 DARPins and 11 GFPs were removed from the refined map to generate an asymmetric map that contained only the symmetric core and one DARPin with a bound GFP. This asymmetric map was used to perform signal subtraction on T symmetry expanded particles^27,28^. 3D classification without alignment was performed on the post-signal subtraction particles. Two additional rounds of refinement with local angular searches were performed on the 3D class with the best density for the GFP in RELION 3.0. The first round of refinement used a mask that contained the symmetric core and one DARPin with a bound GFP. The second round used a mask containing only one DARPin and its bound GFP, and one trimer of each type adjacent to them. The refined particles were subjected to a final round of multi-body refinement^36^. We defined two bodies, one being the symmetric core and the other being the density enclosed by the mask used in the second round of refinement. The data processing procedure is outlined in Supplementary Figure 2. The local resolution of the final map was established using the Resmap program^29^. The median and quartile values for the local resolution for the Darpin component and for the GFP component are based on local resolution values sampled in increments of 0.25 Å using Resmap and analyzed over grid points surrounding the protein molecules (less than 3 Å from the center of an atom belonging to the specified protein component).

All coordinate refinements were performed with phenix.real_space_refine^37^. For the symmetric core, the atomic coordinates of our previously determined cryo-EM structure (PDB 6C9I)^11^ were fitted into the cryoSPARC homogeneous refinement map in Chimera^35^ and allowed residues to be refined individually against this map. Additional residues in the DARPin region were built based on the previously determined DARP14 structure (PDB 6C9K)^11^. For the model including the whole DARPin sequence, the body 1 map from multi-body refinement in RELION was sharpened with phenix.auto_sharpen^37^ with half-map based resolution-dependent local sharpening and used for model building. The residues in 6C9K were corrected to match those in 3G124 and the added Mut5 mutations. The model was then trimmed to contain one trimer of subunit A, one trimer of subunit B, and one DARPin. The trimmed model was fitted into the sharpened map and refined iteratively with secondary structure restraints while allowing morphing or refinement at individual residues. Output from phenix was inspected manually and edited in COOT^38^. Next, to properly fit a sfGFP structure (PDB 4W6B)^39^ into the GFP density of the same map, we utilized an existing crystal structure –a complex between DARPin and eGFP (PDB 5MA8)^20^. After aligning DARPin to DARPin in the DARP14-3G124Mut5 and 5MA8 structures, and replacing the eGFP in 5MA8 with the sfGFP in 4W6B, the sfGFP model was further refined with rigid body movement. The quality of refined models were assess with EMRinger^40^. Note that the EM-Ringer score (about 2.0) puts the GFP in the same range as previous structures reported at resolutions of 3.8 Å or better.

### Reporting summary

Further information on research design is available in the Nature Research Reporting Summary linked to this article.

## Supplementary information

Supplementary Information

Description of Additional Supplementary Files

Supplementary Movie 1

Reporting Summary

## Data Availability

The data supporting the findings of this manuscript are available from the corresponding author upon reasonable request. A reporting summary for this Article is available as a Supplementary Information file. Model coordinates and density maps are available in the Protein Data Bank (PDB ID 6NHT, 6NHV) and the EM Data Bank (EMD-9373, EMD-9374).
