# Supplementary material for: A 3.8 Å resolution cryo-EM structure of a small protein bound to an imaging scaffold
Source: Nat Commun. 2019 Apr 23;10:1864. doi: 10.1038/s41467-019-09836-0 (PMC6478846; doi:10.1038/s41467-019-09836-0)
Supplement: Supplementary file 1 — Supplementary Information [file 41467_2019_9836_MOESM1_ESM.pdf]

Supplementary information for:

A 3.8 Å Resolution Cryo-EM Structure of a Small Protein Bound to a Modular Imaging Scaffold

Liu et al.

Supplementary Notes

Protein sequences

- DARP14-3G124Mut5

Subunit A:
MRITTKVGDKGSTRLFGGEEVWKDSPIIEANGTLDELTSFIGEAKHYVDEEMKGILEEIQNDIYKIMGEIGSKG
KIEGISEERIAWLLKLILRYMEMVNLKSFVLPGGTLESAKLDVCRTIARRALRKVLTVTREFGIGAEAAAYLLALS
DLLFLLARVIEIEQGKKLLEAARAGQDDEVRILMANGADVNAADDVGVTPLHLAAQRGHLEIVEVLLKCGAD
VNAADLWGQTPLHLAATAGHLEIVEVLLKNGADVNARDNIGHTPLHLAAWAGHLEIVEVLLKYGADVNAQ
DKFGKTPFDLAIDNGNEDIAEVLQKAA

Subunit B:
MPHLVIEATANLRLETSPGELLEQANKALFASGQFGEADIKSRFVTLEAYRQGTAAVERAYLHACLSILDGRDI
ATRTLLGASLCAVLAEAVAGGGEEGVQVSVEVREMERLSYAKRVVARQRLEHHHHHH

- Super folder GFP V206A

MSKGEELFTGVVPILVELDGDVNGHKFSVRGEGEGDATNGKLTLKFICTTGKLPVPWPTLVTTLTYGVQCFSR
YPDHMKRHDFFKSAMPEGYVQERTISFKDDGTYKTRAEVKFEGDTLVNRIELKGIDFKEDGNILGHKLEYNFN
SHNVYITADKQKNGIKANFKIRHNVEDGSVQLADHYQQNTPIGDGPVLLPDNHYLSTQSALSKDPNEKRDH
MVLLEFVTAAGITHHHHHH

Primer sequences

- sfGFP V206A quickchange

5’-CCTGTCGACACAATCTGCGCTTTCGAAAGATCC-3’

5’-GGATCTTTCGAAAGCGCAGATTGTGTCGACAGG-3’

Supplementary Tables

Supplementary Table 1. Cryo-EM data collection, refinement and validation statistics

	Cage core (EMDB-9373) (PDB 6NHT)	Partial core, DARPin, GFP (EMDB-9374) (PDB 6NHV)
**Data collection and processing**		
Magnification	130 kX	130 kX
Voltage (kV)	300	300
Electron exposure (e–/Å^2)	56	56
Defocus range (μm)	-2 to -2.5	-2 to -2.5
Pixel size (Å)	1.07	1.07
Symmetry imposed	T	C1
Initial particle images (no.)	91,809	963,036 (sym. expanded)
Finals particle images (no.)	80,253	91,211 (sym. expanded)
Map resolution (Å)	2.9	3.5
FSC threshold 0.143		
Map resolution range (Å)	2.3-3.5	3.2-5.6
**Refinement**		
Initial model used (PDB code)	6C9I, 6C9K	6C9I, 6C9K, 5MA8, 4W6B
Model resolution (Å)	2.9	3.5
FSC threshold 0.143		
Map sharpening *B* factor (Å^2)	cryoSPARC_v2 auto-sharpening	82.0
Model composition		
Non-hydrogen atoms	26136	8948
Protein residues	3432	1166
Ligands	0	1
B factors (Å^2)		
Protein	49.3	NA
Ligand	NA	NA
R.m.s. deviations		
Bond lengths (Å)	0.006	0.011
Bond angles (°)	0.733	0.945
Validation		
MolProbity score	1.03	1.39
Clashscore	2.27	7.17
Poor rotamers (%)	0.45	0.21
Ramachandran plot		
Favored (%)	96.5	95.3
Allowed (%)	3.5	4.7
Disallowed (%)	0	0
EMRinger score	2.73	Overall 1.82, GFP 1.99

Supplementary Figures

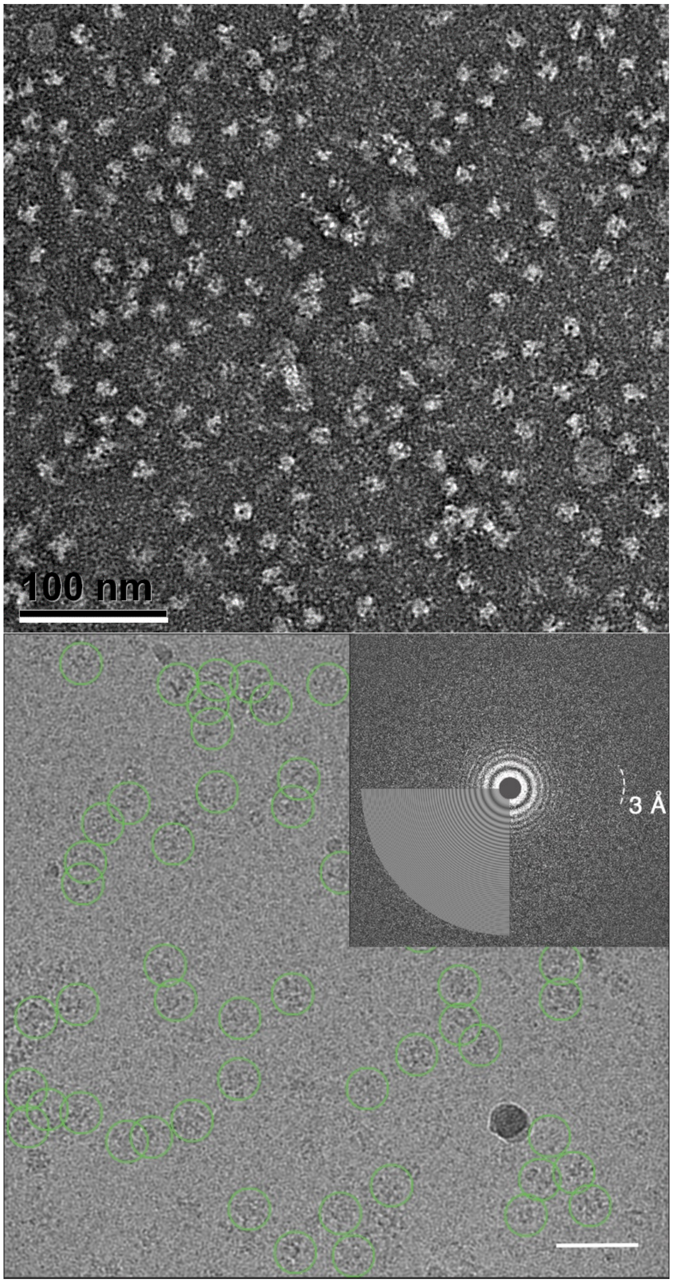

Supplementary Figure 1. (Top) Negative stain image of the scaffold complexed with GFP. Scale bar 100 nm. (Bottom) Motion-corrected, 20 Å low-pass filtered cryo-EM micrograph with inset CTF correction result. Green circles indicate positions of particles. Scale bar, 50 nm.

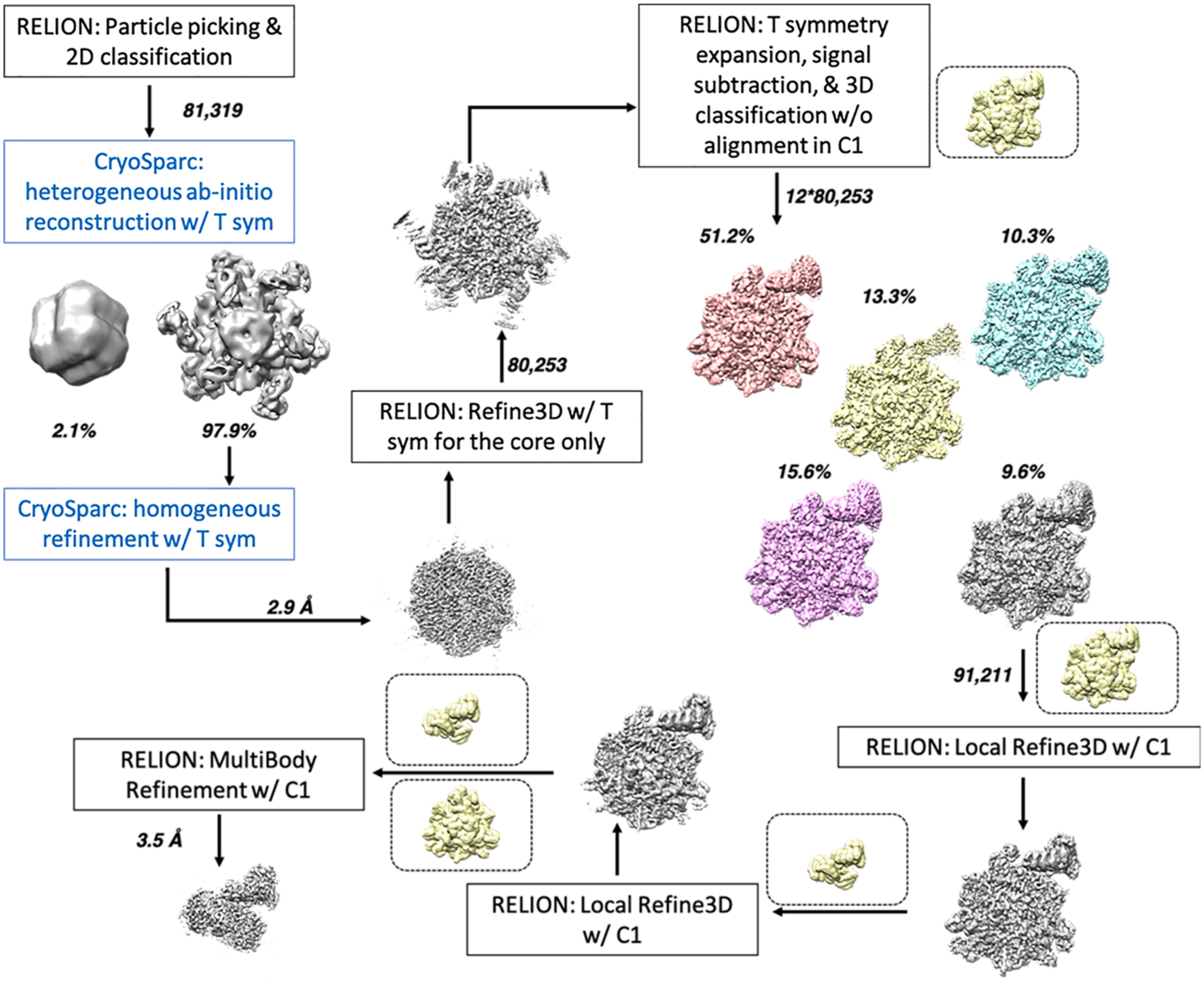

Supplementary Figure 2. Cryo-EM data processing flow chart. Yellow densities inside dotted boxes indicate the masks used. For the 3D classification result, the coloring scheme is: class 1, grey; class 2, yellow; class 3, cyan; class 4, rose; class 5, pink.

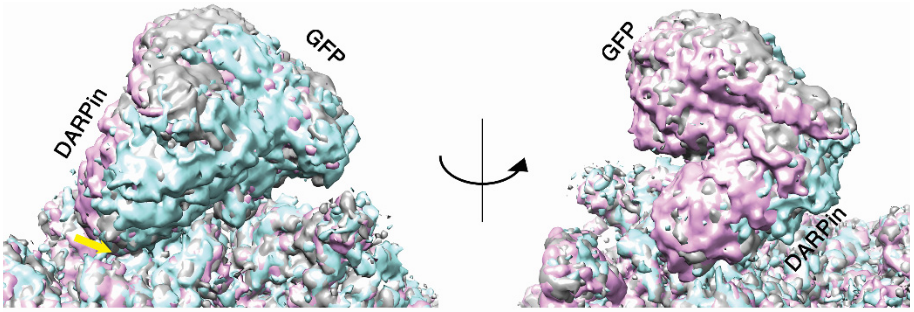

Supplementary Figure 3. Comparisons between 3D classes (class 1, 3, and 5) of signal subtracted particles. Only a portion of the density is shown for clarity. An arrow highlights the secondary contact site between the DARPin (Gly 187) and Gly 108-Thr 109 on subunit A of the core assembly. Each class is colored the same as in Fig. S2.
