# Supplementary material for: A 3.8 Å resolution cryo-EM structure of a small protein bound to an imaging scaffold
Source: Nat Commun. 2019 Apr 23;10:1864. doi: 10.1038/s41467-019-09836-0 (PMC6478846; doi:10.1038/s41467-019-09836-0)
Supplement: Supplementary file 2 — Description of Additional Supplementary Files [file 41467_2019_9836_MOESM2_ESM.pdf]

Description of Additional Supplementary Files

File Name: Supplementary Movie 1
Description: Cryo-EM density map and atomic model of the GFP cargo protein
